# Identification of precursor transcripts for 6 novel miRNAs expands the diversity on the genomic organisation and expression of miRNA genes in rice

**DOI:** 10.1186/1471-2229-8-123

**Published:** 2008-12-02

**Authors:** Séverine Lacombe, Hiroshi Nagasaki, Carole Santi, David Duval, Benoît Piégu, Martine Bangratz, Jean-Christophe Breitler, Emmanuel Guiderdoni, Christophe Brugidou, Judith Hirsch, Xiaofeng Cao, Claire Brice, Olivier Panaud, Wojciech M Karlowski, Yutaka Sato, Manuel Echeverria

**Affiliations:** 1 Laboratoire Génome et Développement des Plantes, UMR5096, Université de Perpignan via Domitia – CNRS-IRD, 52, Av. Paul Alduy, 66860 Perpignan Cedex, France; 2 Graduate School of Bioagricultural Sciences, Nagoya University, Chikusa, Nagoya 464-8601, Japan; 3 DAP, UMR 1098, Université de Montpellier II-CIRAD-INRA-SUPAGRO 2477 Avenue Agropolis, F-34398 Montpellier Cedex 5, France; 4 INRA-SUPAGRO, UMR BGPI, Campus Baillarguet, F-34398 Montpellier Cedex 05, France; 5 State Key Laboratory of Plant Genomics and National Center for Plant Gene Research, Institute of Genetics and Developmental Biology, Chinese Academy of Sciences, Beijing 100101, PR China; 6 Bioinformatic Laboratory, A. Mickiewicz University, Umultowska 89, 61-614, Poznan, Poland

## Abstract

**Background:**

The plant miRNAs represent an important class of endogenous small RNAs that guide cleavage of an mRNA target or repress its translation to control development and adaptation to stresses. MiRNAs are nuclear-encoded genes transcribed by RNA polymerase II, producing a primary precursor that is subsequently processed by DCL1 an RNase III Dicer-like protein.

In rice hundreds of miRNAs have been described or predicted, but little is known on their genes and precursors which are important criteria to distinguish them from siRNAs. Here we develop a combination of experimental approaches to detect novel miRNAs in rice, identify their precursor transcripts and genes and predict or validate their mRNA targets.

**Results:**

We produced four cDNA libraries from small RNA fractions extracted from distinct rice tissues. By *in silico *analysis we selected 6 potential novel miRNAs, and confirmed that their expression requires OsDCL1. We predicted their targets and used 5'RACE to validate cleavage for three of them, targeting a PPR, an SPX domain protein and a GT-like transcription factor respectively.

In addition, we identified precursor transcripts for the 6 miRNAs expressed in rice, showing that these precursors can be efficiently processed using a transient expression assay in transfected *Nicotiana benthamiana *leaves. Most interestingly, we describe two precursors producing tandem miRNAs, but in distinct arrays. We focus on one of them encoding osa-miR159a.2, a novel miRNA produced from the same stem-loop structure encoding the conserved osa-miR159a.1. We show that this dual osa-miR159a.2-osa-miR159a.1 structure is conserved in distant rice species and maize. Finally we show that the predicted mRNA target of osa-miR159a.2 encoding a GT-like transcription factor is cleaved *in vivo *at the expected site.

**Conclusion:**

The combination of approaches developed here identified six novel miRNAs expressed in rice which can be clearly distinguished from siRNAs. Importantly, we show that two miRNAs can be produced from a single precursor, either from tandem stem-loops or tandemly arrayed in a single stem-loop. This suggests that processing of these precursors could be an important regulatory step to produce one or more functional miRNAs in plants and perhaps coordinate cleavage of distinct targets in the same plant tissue.

## Background

In eukaryotes microRNAs (miRNAs) are single stranded 20–22 nucleotide RNAs that direct silencing of an mRNA target by an RNA Inducing Silencing Complex (RISC). In plants most miRNAs have extensive complementarity to their target mRNA and induce its cleavage or translational inhibition. Many of the targets encode transcription factors or signalling proteins controlling different steps of plant development. In addition, miRNAs have also been identified that respond to nutrient availability or abiotic stress, suggesting an important role in plant adaptive responses. Furthermore, by targeting genes encoding proteins of the miRNA machinery, they regulate their own biogenesis and function [for review see [1]].

MiRNAs are structurally related to the more abundant short-interfering RNAs (siRNAs) but can be clearly distinguished by their biogenesis pathway [2]. In *Arabidopsis*, it has been shown that most miRNAs are encoded by independent genes transcribed by RNA polymerase II, producing a primary precursor (pri-miRNA) characterised by a stem-loop structure containing the miRNA. Genetic analysis has shown that the pri-miRNA is processed by the RNase III Dicer-like enzyme 1 (DCL1) to liberate miRNA/miRNA* (the asterisk denotes the sequences coded on the opposite strand to the miRNA in the stem part of its precursors) duplexes from the stem structure. The miRNAs are selectively loaded into the RISC whereas the miRNAs* are subjected to degradation [1,2].

Identification of miRNAs in different plant species has been based on the cloning of small RNA fractions and/or the prediction of miRNA genes in genomic sequences based on sequence similarity [1]. These approaches have revealed or predicted several hundred miRNA genes in *Arabidopsis*, rice and poplar, for which complete genomes are available. All the plant miRNAs reported are available in the Sanger miRNA register [3].

At present in rice there are 240 miRNAs distributed in 62 families [4] reported in the Sanger miRBase Register [3]. Among them, only 20 families are conserved in *Arabidopsis *and poplar, while the others are specific to monocots or rice. Most conserved miRNAs are encoded by multigene families producing identical or very similar miRNAs with a high level of expression. The conserved miRNAs were selected early in evolution and have important functions for plant development or adaptation to the environment [5,6]. The non-conserved miRNAs are usually encoded by single loci and have low levels of expression. They appeared more recently in evolution and their function as canonical miRNAs has not yet been demonstrated [7].

Recently, high-throughput sequencing applied to small RNAs in *Arabidopsis *[7,8], rice [9-11] and other plant species [12-14] have greatly increased the number of miRNA candidates. These data revealed many new “potential” miRNA gene families in the different species but most of them were non-conserved phylogenetically. Nevertheless, in the absence of available DCL1 mutants or any functional test, some of these novel potential miRNAs could be difficult to distinguish from the abundant siRNAs that derive from repeated or retroelement sequences or from complementary antisense RNAs [9,15].

In our laboratory we proposed to identify and characterise novel miRNAs that control development in rice and monocots. To this aim, we produced four cDNA libraries derived from different rice tissues that were sequenced and analysed *in silico *to identify potential miRNA genes. These were then experimentally tested by identification of their precursor miRNAs transcripts and dependence of their expression on rice OsDCL1, an Arabidopsis DCL1 homolog in rice which is essential for miRNA biogenesis. Using these criteria, we have identified six novel miRNAs expressed in rice. We predict their mRNA targets and, for three of them, demonstrate cleavage at the predicted target site *in vivo*, suggesting their implication in an organellar function, phosphate homeostasis and regulation of a GT-like transcription factor respectively. Finally we provide two examples of genes producing a single precursor encoding two miRNAs, indicating that plant miRNA precursors have the potential capacity to encode several miRNAs and perhaps coordinate their expression.

## Results

### Identification of 6 novel rice miRNAs

Four independent cDNA libraries were generated from the 19–30 nucleotide small RNA fractions extracted from roots, seedlings, panicle and leaves (see methods). About 2500 clones were sequenced from each library, producing overall 10,000 sequences. The sequences were first "cleaned" to discard rRNAs, snoRNAs tRNAs and chloroplastic RNA contaminants. The remaining 1495 non redundant sequences were aligned using BLASTN with genomic sequences from *Oryza sativa *ssp. *Japonica *cv Nipponbare anotated in TIGR version 4 of rice genome annotation and subsequently actualised in the IGSRP built 4 from Rice Annotation Project Database RAP-DB [4]. Only sequences giving perfect matches to the genome were retained. These were analysed for potential stem-loop structures harbouring the miRNA/miRNA* duplex that could represent the precursor pre-miRNA [1]. Sequences mapping to highly repeated sequences annotated in RAP-DB [4] and the RetroOryza database for rice retroelements [16] were discarded as they probably represent siRNAs [9,15]. We also discarded most sequences matching protein-coding genes as they could represent mRNA degradation products. An exception was osa-miR2055 which is encoded in an ORF but whose expression was found to depend on OsDCL1 (see below). From this analysis we obtained 69 putative miRNAs mapping to different gene loci. Among these, 45 (65%) correspond to conserved miRNAs previously described in *Arabidopsis thaliana *and/or *Oryza sativa *or are new paralogues of already described families. Many of them were cloned multiple times in the different libraries. The most abundant were osa-miR169, encoded by a large multigene family, which was cloned 117 times and osa-miR168 that we cloned 40 times (result not shown). This is in agreement with the work that used 454 pyrosequencing to identify small RNAs [11] Altogether our results on the conserved miRNAs is in good agreement with previous and recent reports on rice miRNA distribution and validate our cloning procedure and the quality of our library.

We also cloned three distinct classes of 21 nucleotide small RNAs derived from predicted stem-loop structures encoding conserved (canonical) miRNAs. The first category corresponds to osa-miR156h*, osa-miR393b* and osa-miR396d*. The second corresponds to a novel RNA that has a 10 nucleotide overlap with osa-miR393 (not shown), highly similar to the situation reported for the osa-miR444 family [10,11]. The third case corresponds to osa-miR159a.2 (Table 1), a novel miRNA derived from the same precursor encoding osa-miR159a.1 (described in details below)

**Table 1:** Novel miRNA candidates

**miRNAs**	**Sequence (5'-3')**	**Size**	**N° loci**	**Frequency**	**Locus**	**FL cDNA**	**Conservation**
miR1425	UUAGGAUUCAAUCCUUGCUGC	21	1	x2 (s)	Intergenic	yes	-
miR1428e	UAAGAUAAUGCCAUGAAUUUG	21	11	x2 (s)	Intergenic	yes	-
miR2055	UUUCCUUGGGAAGGUGGUUUC	21	2	x1 (s)	Exon (s)	yes	-
miR827a	UUAGAUGACCAUCAGCAAACA	21	1	x1 (l)	Intergenic	no	dicots
miR1874	UAUGGAUGGAGGUGUAACCC	20	1	1 × 1 (i)	Intergenic	no	-
miR159a.2	UUGCAUGCCCCAGGAGCUGCA	21	1	x2 (s)	Intergenic	yes	monocots

In addition to osa-miR159a.2 we identified five other novel miRNA candidates that had not been described in any previous screening (Table 1). The osa-miR1425 and osa-miR1428e were cloned twice in the seedling and panicle libraries respectively, while the 3 others were cloned only once. Only osa-miR827a has an homolog in *Arabidopsis thaliana*, corresponding to ath-miR827, recently identified by high-throughput sequencing [7,8], but with a two nucleotide mismatch. The other miRNAs, with the exception of osa-miR159a.2 which is found in maize (see below), seem specific to rice. We did not find homologues in the other monocots but this is not conclusive as complete genomic sequences are not available for the other monocots.

Altogether we identified six novel miRNA candidates in rice that we named osa-miR1425 to osa-miR159a.2. While this manuscript was in preparation, high-throughput sequencing also identified osa-miR1425 but these was not described further [10,11]. We present here a detailed analysis for each of the novel miRNA identified.

### Expression profile of novel miRNAs

**Figure 1:**
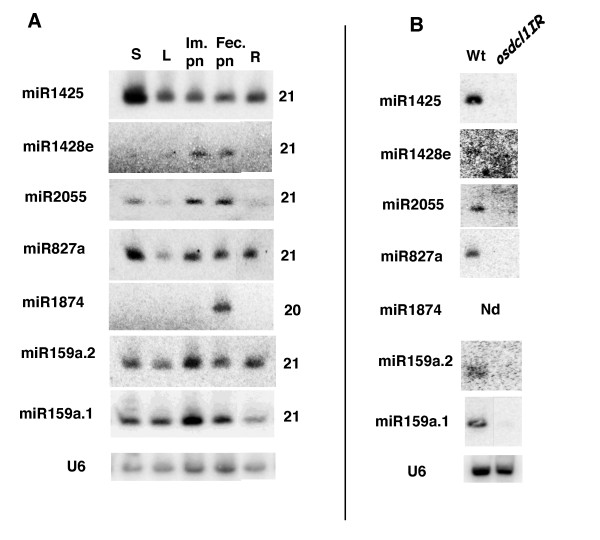
**Expression profile of novel miRNA candidates and dependence on OsDCL1**. A) Northern blot analysis was carried on using total RNAs from the indicated tissues and complementary probes to the candidate miRNAs (see methods). A control for expression of conserved osa-miR159a was also included. S, seedlings; L, mature leaves; Im. pn, immature panicles; Fec. Pn: panicles after fertilisation; R, roots. U6 is a control for RNA loading made with a probe complementary to U6 snRNA from Arabidopsis which is conserved in all plants. B) Northern blot analysis for different miRNA candidates was carried on total RNA extracted from rice seedling from wild type and *osdcl1*IR lines as indicated. Dependence of osa-miR1874, expressed only in panicles, could not be tested in the *osdcl1*IR lines as these have low fertility and produce small panicles.

We analysed the expression profiles of the six novel miRNAs by Northern blot analysis in different rice tissues (Figure 1A). A control for loading is shown with a probe complementary to U6 snRNA. Osa-miR1425, osa-miR2055, osa-miR827a and osa-miR159a.2 were detected in all tissues but with differential accumulation. Osa-miR1428e had lowest level of expression and was preferentially expressed in panicles (inflorescences) while osa-miR1874 was detected only in fertilised panicles.

The miRNAs can be distinguished from repeat-associated siRNAs, which is the most abundant class of small RNAs in plants, by their dependence on DCL1 activity for their biogenesis [1]. To confirm that these are *bona fide *miRNAs, we analysed their accumulation in seedlings from rice *osdcl1*IR RNAi lines with reduced DCL1 activity [17]. Clearly accumulation of all of them, except for osa-miR1874, is drastically reduced in *osdcl1 *IR plants compared to wild type plants (Figure 1B). Accumulation of osa-miR1874 could not be checked in the *osdcl1*IR mutants because these plants produce small panicles, the only stage where osa-miR1874 is expressed. Also in the case of osa-miR1428e this was difficult to assess as this miRNA is expressed mainly in panicles, with very little expression in seedlings (Figure 1B). Overall this analysis confirms that the biogenesis of all our predicted miRNAs, except perhaps for osa-miR1874 that could not be tested, depends on OsDCL1 as expected for canonical miRNAs.

### Identification and characterisation of miRNA precursor transcripts and genes

Remarkably, little information has been produced on miRNA precursors in rice although this is essential to define a miRNA gene and confirm its expression. Therefore, we first searched for miRNA precursors among the cDNA and EST libraries available in public databases by alignment of our candidate miRNAs against them.

### osa-miR1425, a canonical miRNA gene structure

**Figure 2:**
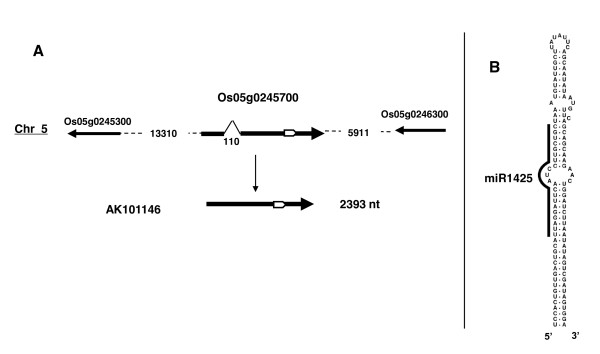
**osa-miR1425 gene and transcript precursor**. A) The Os05g0245700 gene and the corresponding cDNA. The intron position and size are indicated. The white arrow in the gene and the cDNA indicate the predicted stem-loop drawn in B. Flanking genes and their predicted sense of transcription are indicated by arrows (size of arrows are not drawn to scale). Distance to annotated flanking genes is indicated in nucleotides. B) Predicted stem-loop structure [48]. The miRNA is indicated by the bar.

Osa-miR1425 is encoded by a single locus (Os05g0245700) producing a 2393 nucleotide cDNA (AK101146) (Figure 2A). This cDNA has little coding capacity with the biggest ORF predicted to encode a 78 amino acid protein with no similarity to any other protein. A stem-loop structure with the miRNA/miRNA* can be predicted at the 5'end of the cDNA (Figure 2B). It is highly likely that this long cDNA produced by the Rice Full length cDNA Consortium [18] corresponds to the primary precursor pri-osa-miR1425.

### osa-miR1428e, a tandem miRNA gene precursor

**Figure 3:**
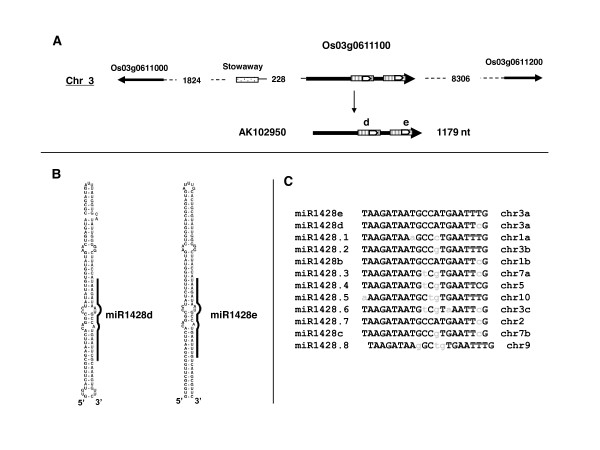
**osa-miR1428e gene and transcript precursor**. A) Annotations similar to Figure 2. Dotted box indicates a Stowaway retroelement. The hatched boxes indicate repeated sequenced in the mRNA including the two predicted stem-loop structures containing osa-miR1428e identified in our library, and the predicted osa-miR1428d. B) Stem-loop predicted structures for osa-miR1428e and osa-miR1428d found in AK102950. C) Alignment of osa-miR1428 isoforms from the different loci. Nucleotides in small cases indicate divergence related to the cloned osa-miR1428e sequence. osa-miR1428e and osa-miR1428d derive from the same locus. The miR1428.1 to miR1428.7 refer to predicted miRNAs not annotated in Sanger database. The nomenclature chr3a, chr3b, etc... refer to different loci in the same chromosome. The detailed chromosomal position is given in methods.

The cloned osa-miR1428e is encoded by a single locus (Os03g0611100) on chromosome 3 which gives a 1179 nucleotide mRNA (AK102950) with no protein coding capacity. Remarkably, this mRNA has two osa-miR1428 stem-loops in tandem (Figure 3A). The cloned sequence corresponds to osa-miR1428e produced from the second stem-loop and differs by one nucleotide from osa-miR1428d (Figures 3B &3C).

In addition to the tandem osa-miR1428e locus, we found 10 other loci encoding osa-miR1428 homologs (Figure 3C) flanked by highly conserved genomic regions including and extending beyond the predicted stem-loop structures (not shown). Remarkably none of the additional osa-miR1428 loci had a tandem osa-miR1428 stem-loop organisation. The high sequence conservation of these osa-miR1428 loci suggests that they could be functional. Recently three of these loci have been found expressed, encoding osa-miR1428a, osa-miR1428b and osa-miR1428c [10,11]. Most of others are located in retroelement-rich regions for which there is no indication of expression, as no cDNA or EST has been reported from them. The only exception is osa-miR1428.1 which maps to an intron of gene Os03g0791800 which gives an mRNA (AK067703) coding for a conserved ubiquitin-carrier protein. Remarkably, the host intron for osa-miR1428.1 is 3531 nucleotides which is much larger than the 150–200 nucleotide average for plant introns [19]. We do not know whether the intronic osa-miR1428.1 is produced *in vivo*, as is the case for several intronic miRNAs [1]. In any case, the cloned osa-miR1428e can be distinguished from all other osa-miR1428 isoforms by one or two nucleotide differences (Figure 3C), clearly indicating that it derives by processing from the osa-miR1428 tandem miRNA precursor.

Finally we also identified by BLASTN analysis a nearly identical tandem osa-miR1428 gene conserved in *Oryza sativa *ssp *indica*, another rice subspecies, showing that the osa-miR1428 tandem miRNA gene is conserved and could have an important function in rice (not shown).

### osa-miR2055, a complex genomic organisation

**Figure 4:**
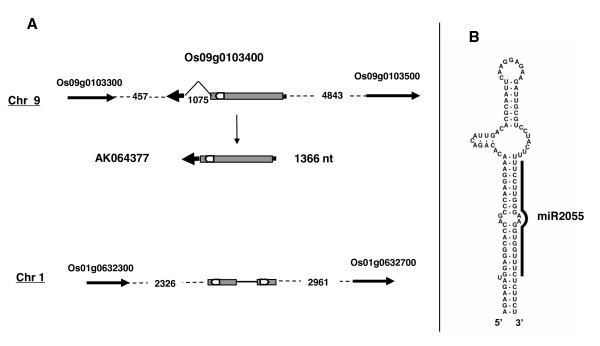
**osa-miR2055 gene and transcript precursor**. Annotations similar to Figure 2. The osa-miR2055 predicted stem-loop is shown by open arrow in chromosome 9 and on the cDNA. The grey box indicates a predicted ORF in Os09g0103400 gene. Part of the ORF sequences and the stem-loop are found repeated in chromosome 1, as indicated.

The osa-miR2055 sequence maps to two genomic loci (Figure 4A). The first is in the first exon of gene Os09g0103400, which gives a 1366 nucleotides cDNA (AK064377). This cDNA is predicted to encode a 331 amino-acid hypotethical protein with no similarity to any known protein or protein motif. A predicted stem-loop encoding osa-miR2055/osa-miR2055* duplex is found in the ORF region (Figure 4B).

A nearly identical sequence to the pre-osa-miR2055 stem-loop is found duplicated in an intergenic region of chromosome 1, with no predicted gene. Alignment of these sequences reveals that the pre-osa-miR2055 stem-loop is part of an inverted duplication in chromosome 1 which is conserved in Os09g0103400 and the expressed mRNA (Figure 4A). We used RT-PCR with specific primers to detect expression of the chromosome 1 pre-osa-miR2055 locus in rice seedlings. This revealed the expected RT-PCR amplification product which was confirmed by sequencing (not shown). This indicates that the second locus on chromosome 1 is transcribed and could also produce osa-miR2055.

### Identification of osa-miR827a and osa-miR1874 intermediate precursors

**Figure 5:**
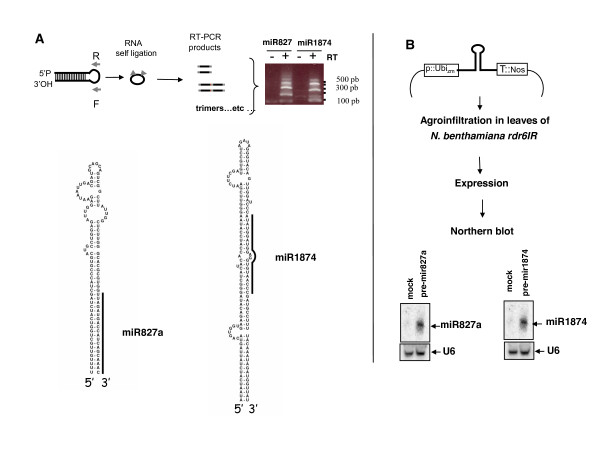
**Identification and validation of osa-miR827a and osa-miR1874 transcript precursors**. A) Schematic for cloning of pre-osa-miR827a and pre-osa-miR1874 by cRT-PCR. The sequence was cloned by cRT-PCR from total RNA extracted from rice seedlings, as indicated in methods. Arrows indicated by R and F refers to primers for PCR amplification steps. The gel shows analysis of of cRT-PCR amplified products products. - and + refers to absence or presence of reverse transcription previous to PCR steps, as a control for DNA contamination. B) The cloned cRT-PCR products and folding into stem-loop structures. 5' and 3' end indicates the extremities of the cloned products. C) Transient expression of pre-osa-miR827a and pre-osa-miR1874 in *Nicotiana benthamiana *leaves. The predicted stem-loop encompassing miR827a or miR1874 plus 150 nucleotides of 5' and 3' sequences was expressed from pCUbi vector in *Nicotiana benthamiana *IR lines leaves, as schematised. P::Ubi_zm _indicates maize Ubiquitin promoter and T:: Nos indicates Nopaline synthetase polyA site. The products were analysed by Northern blot using oligonucleotide probes complementary to osa-miR827a or osa-miR1874. Mock is a control of leaves transfected with an empty pcUbi vector.

Osa-miR827a is encoded by a single locus mapping to an intergenic region on chromosome 2 (see Additional file 1). No cDNA or EST is reported for this genomic region. Therefore we used circular RT-PCR (cRT-PCR) [20] to clone a precursor transcript from a total RNA fraction extracted from rice leaf tissue (Figure 5A). We obtained several clones, all corresponding to a sequence of 116 nucleotides that folds into a stem-loop structure (Figure 5B). Our observation that rice miRNA precursors are encoded by large transcripts suggests that the cloned sequence represents an intermediate precursor corresponding to pre-osa-miR827a. We were nevertheless unable to clone a larger product by cRT-PCR, perhaps due to rapid processing or to a low level of accumulation of pri-miRNA produced from this gene.

We confirmed that the genomic predicted stem-loop region including the cloned pre-osa-miR827a is processed to mature osa-miR827a by transient expression of *Arabidopsis *miRNA precursors in *Nicotiana benthamiana rdr6IR *lines (Figure 5C) [21]. Expression in *rdr6 IR *line plants is necessary to reduce production of siRNAs from transfected stem-loop regions. We adapted this assay to express rice pre-miRNA using the pCUbi:Nos expression vector that drives transgene expression from a maize ubiquitin promoter which is constitutively expressed both in dicotylodenous and monocotyledonous plants [22]. Thus this construct could subsequently be used to transient overexpression of the rice miRNA. First we verified that a canonical rice miR164a precursor was expressed in *Nicotiana benthamiana *leaves, producing miR164 that could be detected by Northern blot with a specific probe while no signal was detected using probes complementary to other regions of the stem-loop pre-miR164 (data not shown).

We then used this assay to test whether the 507 base pair genomic region encompassing the cloned pre-osa-miR827a sequence could be processed into osa-miR827a and detected using a probe complementary to osa-miR827a. Clearly the result shows that a specific signal of 21 nucleotides is detected in the transfected leaves, which is absent in leaves transfected with an empty vector (Figure 5C). This confirms that osa-miR827a is produced from this genomic locus *in vivo*.

The osa-miR1874 sequence maps to a single locus in an intergenic region between two distant genes (see Additional file 1). No cDNA or EST has been reported for this locus. Again we used cRT-PCR to clone the pre-osa-miR1874 from RNA fraction extracted from rice mature panicles. The cloned pre-osa-miR1874 is 150 nucleotides long and perfectly folds into a predicted stem-loop (Figure 5B). Probably, this represents one of the last steps of processing from a pri-osa-miR1874 precursor, as for pre-osa-miR827a. Using the agroinfiltration assay in *Nicotiana benthamiana*, we confirmed that the 537 base pair genomic fragment including the cloned pre-osa-miR1874 is sufficient to produce osa-miR1874 (Figure 5C).

### osa-miR159a.2, a single precursor can produce two distinct miRNAs

**Figure 6:**
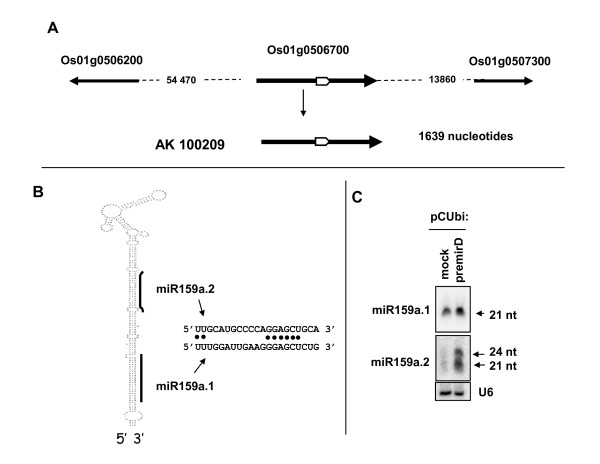
**osa-miR159a.2 gene and transcript precursor**. A) Genomic organisation of the osa-miR159a.2-miR159a.1 B) Stem-loop predicted structure encoding osa-miR159a.2 and osa-miR159a, indicated by bars. The corresponding mature miRNA sequences are indicated by the arrows. C) Expression of pre-osa-miR159a.2 genomic sequence in *Nicotiana benthamiana *leaves. Detection of osa-miR159a.1 and osa-miR159a.2 was done using specific probes. Detection of a miR159 signal in mock transfected leaves is due to endogenous miR159 from *Nicotiana benthamiana*.

The osa-miR159a.2 sequence maps to a single locus in gene Os01g0506700 encoding the conserved osa-miR159a.1. This gene produces a 1653 mRNA (AK100209), probably corresponding to the primary pri-miR159a.1. The osa-miR159a.2 is found in the stem-loop region encoding osa-miR159a.1 but is 21 nucleotides upstream and it has a totally different sequence (Figure 6A and 6B).

In rice the conserved osa-miR159a.1 is part of a multigenic family including six members. Alignment shows that the osa-miR159a.2 sequence has perfect match only with the osa-miR159a.1 gene hairpin locus and can be distinguished from all other osa-miR159 genes by two or three nucleotide differences (not shown). Northern blot analysis reveals that osa-miR159a.2 has the same pattern of expression as osa-miR159a.1 and accumulates to a comparable level (Figure 1A). Indeed the expression level of osa-miR159a.1 detected by Northern blot is over-evaluated as the anti-sense miR159a.1 probe probably cross-hybridises with other members of this family. Finally, as expected for miRNAs, osa-miR159a.2 accumulation in rice depends on OsDCL1 (Figure 1B).

Next we confirmed that the stem-loop region including osa-miR159a.1 could be processed to produce osa-miR159a.2 *in planta *(Figure 6C) using the agro-inoculation assay previously described (Figures 5C). To this aim *Nicotiana benthamiana *leaves were transfected with a construct expressing a 446 nucleotide genomic sequence encompassing the stem-loop encoding both osa-miR159a.1 and osa-miR159a.2. Northern blot analysis with a miR159a.1 complementary probe clearly detected a stronger signal than that detected in leaves transfected with an empty vector (Figure 6C). The positive signal detected for osa-miR159a.1 in control leaves is probably due to cross-hybridisation of the probe with endogenous Nicotiana miR159 which is conserved in plants. These results indicate that rice osa-miR159a.1 is processed from a stem-loop precursor in the Nicotiana leaves.

The osa-miR159a.2 complementary probe clearly detected a strong signal of 21 nucleotides in infiltrated leaves, which is much stronger than the background detected in leaves transfected with an empty vector (Figure 6C). An additional signal of 24 nucleotides was also detected with this probe that was not detected using the osa-miR159a.1 probe (Figure 6C) or when expressing other pre-miRNAs (Figure 5C). Considering that this 24 nucleotide RNA is normally not detected in rice plants (Figure 1A) this could suggest that processing of the osa-miR159a.1 precursor to produce osa-miR159a.2 is not accurate in a heterogeneous system or could implicate another Dicer distinct from DCL1 under our conditions, in which this stem-loop is overexpressed in *Nicotiana benthamiana *leaves.

**Figure 7:**
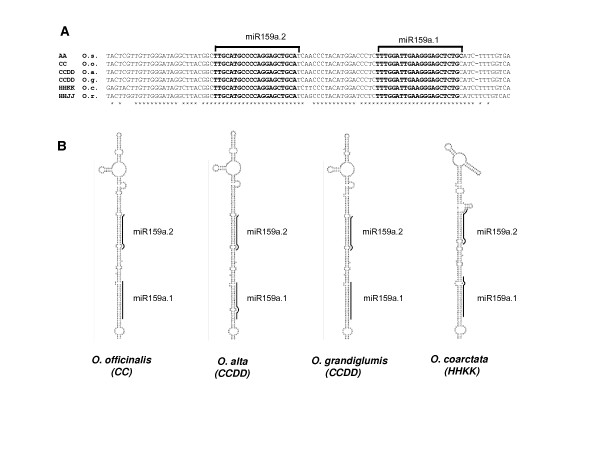
**osa-miR159a.2 conservation in distinct rice species**. A) Alignment of genomic sequences corresponding to stem-loop osa-miR159a.2-miR159a.1 regions from the different rice species obtained as indicated in methods. O.s. *Oryza sativa *genome AA; O.o.; *Oryza officinalis*, genome CC; O.a. alta, genome CCDD; O.g., *Oryza grandiglumis*, genome CCDD; *Oryza coartacta*, genome HHKK; *Oryza ridleyi*, genome HHJJ. * indicates conserved nucleotides. B) Stem-loop structures for the 4 indicted rice species. We were unable to clone the 5'end of the stem-loop from *O. ridleyi *and so we do not present its stem-loop structure.

To further support that osa-miR159a.2 has a functional importance for rice we searched for osa-miR159a.2-osa-miR159a.1 tandem structures conserved in more distant Oryza species with different genome types [23]. Because complete genomic sequences for these Oryza species are not available, we aligned by BLASTN the osa-miR159a.1 precursor with BAC ends sequences from 12 Oryza species available in OMAP . This produced the homolog sequences for *Oryza coarctata *with HHKK genome and a partial sequence for *Oryza ridleyi *with HHJJ genome. The additional species, *Oryza officinalis *with CC genome, *Oryza alta *and *Oryza grandiglumis *both with CCDD genomes, were amplified by PCR from genomic DNA using primers designed from conserved sequences in the osa-miR159a.1 precursor (see methods). These fragments were cloned and sequenced. The results clearly show that, as for *Oryza sativa*, that has an AA genome, all have retained a perfect osa-miR159a.2 sequence just upstream from the conserved osa-miR159a.1 (Figure 7A) producing conserved stem-loop structures (Figure 7B).

**Figure 8:**
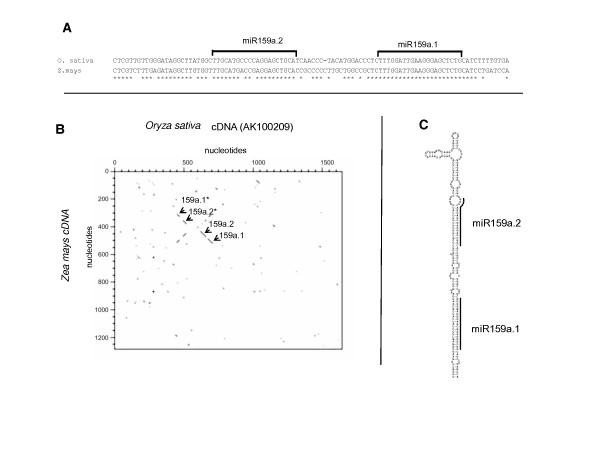
**osa-miR159a.2 homolog in maize**. A) Alignment of *Oryza sativa *and *Zea mays *sequences flanking the conserved miR159a. The position of osa-miR159a.2 and miR159a.1 are indicated. * indicates conserved nucleotides. B) Comparison of osa-miR159a.2-mir159a.1 cDNAs from rice and maize by dot-plot analysis. Dot-plot was made between the *Oryza sativa *full length cDNA 1639 nucleotides (AK100209) and the predicted cDNA from maize derived by contiging maize EST (see methods). The diagonals reveal conserved sequences between maize and rice cDNAs corresponding to mir159a.1, osa-miR159a.2, osa-miR159a.2* and miR159a.1* as indicated by the arrows. C) Stem-loop structure predicted for the *Zea mays *osa-miR159a.2-mir159a.1 precursor.

We looked for osa-miR159a.2 conservation in other monocots. Although miRNA precursor sequences show high divergence except for miRNAs and miRNAs* regions, we found a predicted homolog for osa-miR159a.2 with 2 mismatches in maize located 21 nucleotides upstream from the predicted maize miR159 (Figure 8A). It is important to note that this homology among these two cDNA is restricted to the region encompassing the two miRNAs. This is clearly shown by dot plot analysis that shows four small diagonal fragments at the 5' end of the cDNAs from both maize and rice (Figure 8B). The maize sequence folds perfectly into a predicted stem-loop, similar to the rice osa-miR159a.2-miR159a.1 stem-loop precursors (Figure 8C).

Altogether these results indicate that osa-miR159a.2 is conserved in all Oryza species and in maize, being produced by processing of the osa-miR159a.2-miR159a.1 tandem precursor.

### Target genes

To predict target genes, we carried out a screening considering basic criteria reported for miRNA:mRNA target functional interactions defined in plants as indicated in methods [24,25].

**Figure 9:**
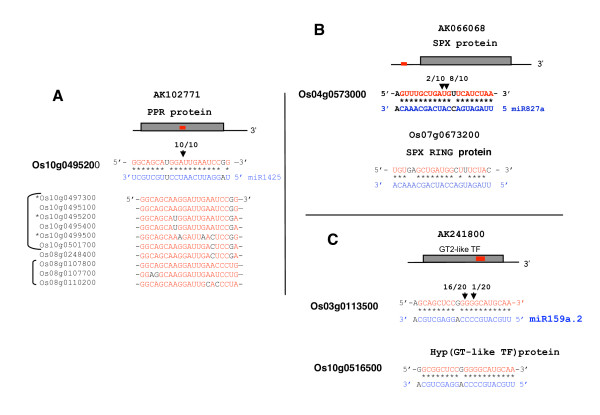
**Validation of targets of novel miRNAs by 5'RLM-RACE**. A) Validation of targets for osa-miR1425. Position of the miRNA complementary site in the mRNA is indicated by the bar. Open box indicates ORF. Arrows indicate cleavage the 5' termini of miRNA-target cleavage products. 8/10 and 2/10 refer to the frequency of clones sequenced. Additional predicted target sites on the other PPR genes are shown. * indicate target validated by Lu et al [10]. B) Validation of targets for osa-miR827a. C) Validation of targets for osa-miR159a.2.

For osa-miR1425, we found numerous predicted mRNAs targets, which all encode PPR proteins (Figure 9A). These are RNA binding proteins characterised by pentatricopeptide motifs and most are addressed to mitochondria or chloroplast in plants [26]. We confirmed cleavage of one PPR mRNA target (Os10g0495200) in rice seedlings *in vivo *by 5'RACE. In addition, two other PPR predicted targets of osa-miR1425 were also recently validated by Lu et al [10]. The other PPR mRNA predicted targets show perfect alignment with these three validated target genes at the osa-miR1425 recognition sites and are therefore also potential targets for osa-miR1425 cleavage (Figure 9A). We note that the five target genes are clustered in a 316 kilobase region on chromosome 10 (Figure 9A). A similar situation is observed for the target PPR genes predicted on chromosome 8, in which 3 out of 4 targets are clustered in a 160 kilobase region (Figure 9A).

The osa-miR827a has two predicted targets encoding two distinct proteins characterised by an SPX domain. This domain is associated with proteins that have been implicated in transport or sensing of phosphate or nitrogen in Arabidopsis [27,28]. We confirmed by 5'RACE cleavage of the 5'UTR of the mRNA corresponding to the best candidate target encoded by Os04g0573000 (Figure 9B).

The osa-miR159a.2 is predicted to target Os03g0113500 that encodes a GT-2 like transcription factor and Os10g0516500 which encodes a hypothetical protein with significant similarity to GT-like transcription factors (Figure 9C). Both genes are expressed, as revealed by corresponding cDNAs in databanks. We carried out 5'RACE analysis to test for cleavage of the GT-2 like transcription factor mRNA *in vivo *and confirmed it by mapping on the predicted miRNA complementary site (Figure 9C).

The osa-miR1428e, osa-miR2055 and osa-miR1874 are predicted to target genes with different functions (see Additional file 2). These predicted targets are all expressed, as demonstrated by the existence of corresponding cDNAs or ESTs in databanks. We could not validate cleavage of predicted targets for osa-miR1428e, osa-miR2055 and osa-miR1874 by 5'RACE. Indeed, this is a common observation for most non-conserved miRNAs, as previously reported by others in Arabidopsis [7] and rice [10]. This could be due to a very low level of expression of the targets or the instability of the cleaved product. For instance the osa-miR1428e target, Os03g0289100, which encodes a predicted protein kinase (see Additional file 2), is expressed but its expression level is very low as revealed by MPSS signatures corresponding to these genes [9]. A similar situation is observed for osa-miR1874, predicted to target Os04g0311100 which encodes a protein of unknown function. Notably MPSS data reveal expression of this gene mainly in ovaries and mature stigmata [9], in agreement with the specific expression of osa-miR1874 in panicles (Figure 1A).

## Discussion

### Identifying novel miRNAs

Using a classical protocol for cloning and sequencing cDNA libraries derived from small RNA fractions we identified 6 novel miRNAs in rice. These 6 novel candidates were selected after a stringent analysis of many thousands of sequences paying particular attention to their precursor RNAs and genomic organisation. Expression of five of them was shown to be dependent on OsDCL1, while the expression of osa-miR1874 could not be tested in *osdcl1*IR mutants (Figure 1B).

Several remarks can be made on our study compared with previous work, including two recent reports using high-throughput sequencing to identify rice small RNAs [10,11]. The first is that our cloning procedure is qualitatively comparable to high throughput sequencing approaches, as we cloned nearly all abundant miRNA species representing conserved miRNA families previously described in *Arabidopsis*, rice or other plant species. The most abundant species in our libraries were osa-miR169 and miR168 that we cloned 117 and 40 times respectively (result not shown). This is in agreement with a recent report using 454 pyrosequencing to identify small RNAs in rice [11] and differs from MPSS high-throughput sequencing that identified osa-miR168 as the most abundant miRNA in their libraries [10].

Remarkably osa-miR827a, a rice miRNA homolog to ath-miR827, had escaped identification by high-throughput sequencing in rice [10,11]. A simple hypothesis is that some conserved miRNAs have low levels of expression which, coupled to a limiting cloning step cannot be compensated by high-throughput sequencing. Overall this work suggests that novel miRNAs, even conserved, should be still identified and expressed in plants under different biological or environmental conditions. We note that an osa-miR827 has been predicted that has 3 mismatches with ath-miR827 [29]. In fact this predicted miRNA maps in a repeated CACTA transposable element (unpublished data). Moreover it is predicted to target an mRNA encoding a sec14 cytosolic factor [29] while both osa-miR827a and ath-miR827 target an mRNA encoding an SPX protein.

A different situation occurs for the non-conserved miRNAs. A few of them which are abundant are regularly cloned, such as osa-miR444 or osa-miR820, that we also cloned. Nevertheless, most of them have a low level of expression and are rarely cloned in more than one library [10,11]. This makes it very difficult to distinguish these non-conserved miRNA from siRNAs that derive from repeated sequences or perfect hairpin structures found in retrosequences. Thus, although we found many candidates fulfilling the miRNAs structural criteria, we retained only those that we could detect by Northern and whose expression is dependent on OsDCL1. The exception was osa-miR1874 for which we could not test OsDCL1 dependence because it is only expressed in panicles, which are extremely reduced in *osdcl1 *IR panicles. Indeed, we believe that a definition of novel miRNA genes will require a deep genomic analysis coupled to identification of precursor miRNAs as reported here.

### The rice miRNA precursors are large transcripts and can encode multiple miRNAs

An important contribution of this work is the identification of precursors for each of the rice predicted miRNAs coupled to a fine analysis on their genomic organisation. Indeed, studies on plant miRNA precursors are limited and mainly restricted to a few examples in Arabidopsis [30,31]. Here we present potential primary precursors for osa-miR1425, osa-miR1428e, osa-miR2055 and osa-miR159a.2 which correspond to long cDNAs reported by the Rice Full length cDNA Consortium [18]. We did not find any reported cDNAs or ESTs for osa-miR827a and osa-miR1874. We believe this is due to low levels of these miRNAs coupled to the rapid processing of pri-miRNA. Intermediate pre-miRNA species seems most stable as we could clone them for osa-miR827a and osa-miR1874 by circular RT-PCR.

In flowering plants several miRNA gene clusters have been reported but most correspond to clusters producing independent transcripts encoding a single miRNAs [32-34]. The large precursors encoding plant miRNAs have the potential to encode multiple miRNAs, either identical or different. Tandem miRNA precursors are common in animals and Chlamydomonas [35] but are rare in moss [36]. In flowering plants only a few cases of tandem miRNAs expressed within a common precursor have been described in detail. These correspond to miR156 and miR395 in rice and maize [37-39] and miR166 in *Medicago truncatula *[40]. These clusters encode similar isoforms from conserved miRNAs. More recently, five tandem miRNA precursor were reported in soybean [41]. Here we provide two new examples of miRNAs gene clusters in rice representing two different cases of precursors encoding tandem miRNAs.

The first corresponds to the Os03g0611100 gene which gives a large precursor encoding tandem copies of osa-miR1428e and osa-miR1428d stem-loop structures (Figure 3). We also identified a nearly identical tandem osa-miR1428 gene in *Oryza sativa *sp *indica *showing that this tandem miRNA gene has been conserved in other rice subspecies (not shown). Thus the osa-miR1428e/d gene is a fourth example in flowering plants producing a tandemly arrayed miRNA precursor but for the first time it concerns a non-conserved miRNA.

The functional significance for expressing tandem array of similar miRNAs is not clear, but it has been shown for the rice/maize miR156 and the Medicago miR166. In the case of the rice osa-miR156 is encoded by 12 loci, but only one of them encodes a tandem MIR156b-156c precursor [37]. This tandem array is functionally important as in maize over-expression of a tandem miR156 gene produces the *Corngrass*1 heterochronic mutant [39]. In the case of the tandem miR166 gene in *Medicago truncatula *it was shown that it is important for the control of root architecture [40].

In the previous examples identical miRNAs were produced from tandem stem-loop structures in a single precursor. The osa-miR159a.2, which is produced from the same stem-loop encoding osa-miR159a.1, represents a different case. In contrast to frequent cases of small RNAs derived from the same precursor encoding the canonical miRNAs that are usually interpreted as slippage of imperfect DCL1 processing [7-11], osa-miR159a.2 is 21 nucleotides from osa-miR159a.1 and has a complete different miRNA mature sequence. The existence of a functional osa-miR159a.2 produced from the osa-miR159a.1 precursor is further strengthened by the perfect phylogenetic conservation of the osa-miR159a.2-osa-miR159a.1 duplex in distant Oryza species and in maize (Figures 7 and 8). Finally, the transient expression of the osa-miR159a.1 stem-loop clearly indicated production of osa-miR159a.2 in agro-infiltrated leaves of *Nicotiana benthamiana*. These observations, coupled to the observation that its predicted target is cleaved *in vivo *(Figure 9) strongly support our hypothesis that osa-miR159a.2 produced from osa-miR159a.1 precursor is a functional miRNA.

Osa-miR159a.2 should not be a unique case and there should be other examples in plants of genes encoding two or more functional miRNAs located in the same stem-loop region. A hint of this situation has already been observed in Arabidopsis and soybean for a few miRNAs [7,8,41]. Expression of these miRNA tandem arrays could represent a mechanism to coordinate the production of various distinct miRNAs to cleave their distinct mRNA targets in the same temporal or developmental stage or tissue. It would be particularly interesting to assess this hypothesis and elucidate the mechanisms involved in the regulation and processing of this stem-loop to generate two miRNAs.

### The functional role of the predicted miRNAs

We confirmed cleavage of predicted targets at the miRNA complementary sites for osa-miR1425, osa-miR827a and osa-miR159a.2 by 5' RACE.

Notably osa-miR1425, corresponding to recently found miRNAs by high-throughput sequencing [10,11], is predicted to target at least 12 genes all encoding PPR proteins. We confirmed cleavage of one of the PPR targets, while cleavage of two other predicted PPR targets was confirmed by others [10]. The PPR are RNA binding proteins characterised by pentatricopeptide motifs and represent one of the largest families in plants including 450 genes in *Arabidopsis *and 477 in rice [26]. This enormous expansion of PPR genes is specific to plants, as in other eukaryotes PPR are encoded by 1 to 6 genes [26]. In plants, most of them are targeted to mitochondria or chloroplasts and have been shown to control virtually all steps of organellar gene expression [26]. The three validated targets plus the 7 other predicted PPR targets of osa-miR1425 (Figure 9A) represent a minor fraction of the 477 rice PPR genes but these genes are clustered on chromosomes 10 and 8. This could be reflected by specific events of duplication unique to this small subset of PPR genes targeted by osa-miR1425. In any case osa-miR1425 would be the first example in eukaryotes of an miRNA controlling organellar biogenesis. It is tantalising to imagine that by altering osa-miR1425 levels in rice one could identify the RNA targets of the PPR proteins, most of which have as yet unknown roles [26].

A distinct case is observed for osa-miR827a. This miRNA is the homolog of recently reported ath-miR827 but differs by two nucleotides [7]. Significantly, both the rice and *Arabidopsis *miR827 are predicted to direct cleavage of mRNAs encoding proteins with an SPX motif. We confirmed cleavage of one of the predicted target at the miRNA complementary site in the 5'UTR by 5'RACE. It has also been shown that the predicted SPX target of ath-miR827 is cleaved *in vivo *at the predicted miRNA complementary site [7]. SPX domains are found in proteins associated with nutrient transport. In *Arabidopsis *they have been found respectively in proteins encoded by PHO and NLA genes controlling adaptation to phosphate [27] and nitrogen [28]. These data strongly suggest a role of osa-miR827a and ath-miR827 in the response of rice and *Arabidopsis *to phosphate or nutrient availability.

We also detected cleavage of osa-miR159a.2, whose predicted target encodes a GT2-like transcription factor. Previously other groups had reported small RNAs produced from stem-loops encoding canonical miRNAs in *Arabidopsis*. Nevertheless, no target cleavage could be shown for any of them and they are probably products of DCL1 slippage during processing of the precursor [7-11]. Therefore, this is a very important result that strongly supports a functional role for osa-miR159a.2, produced from the same stem-loop structure encoding osa-miR159a.1. Interestingly, the second target predicted for osa-miR159a.2 is also related to GT-like transcription factors, in agreement with the observation that some miRNAs target transcription factors from the same family [1]. This also supports the hypothesis that osa-miR159a.2 is a functional miRNA rather than a by-product of DCL1 slippage. Our results suggest a possible significance of co-regulation of TCP and/or MYB genes and GT2-like factors that are targeted by osa-miR159a.1 and osa-miR159a.2, respectively, in rice.

For osa-miR1428e, osa-miR2055 and osa-miR1874 we could not detect any 5'RACE product to confirm cleavage of the predicted target. The lack of cleavage for predicted targets for most reported non-conserved miRNAs is a common observation. This could be due to low levels of expression of mRNA targets, as is the case for osa-miR1428a targets as evaluated by MPSS analysis, or to instability of the cleaved product. An alternative explanation is that some of these miRNA do not direct cleavage of the target but rather control its translation, as recently shown for some plant miRNAs [42] or its expression at another level, for instance at the transcriptional level or by controlling alternative splicing, as shown for some miRNAs in animals [43].

## Conclusion

Overall, we have provided compelling evidence for 6 novel miRNAs in rice, strongly supported by their dependence on OsDCL1. An important point in this analysis is the identification of long pri-miRNA precursors coupled to fine genomic analysis to screen the small RNA sequences. This analysis, even if restricted to a small number of novel candidates, reveals great diversity in miRNA genomic organisation in plants, to which should be added the intronic miRNAs [44] which have not been discussed here. Most importantly, we show two distinct cases of single precursors encoding tandem miRNAs. Our analysis suggest diverse mode of the regulation of expression of miRNA genes and the processing of their miRNA precursors that could be important to coordinate with mRNA target cleavage and on which we know very little in rice and even in other plants.

## Methods

### Cloning of endogenous small RNAs from rice

The rice (*Oryza sativa*) plants used in this study were *japonica cv *Nipponbare. Total RNA was extracted separately from young seedlings, roots, a pool of leaves of two, three and four-week-old plants and a pool of immature, mature panicles and panicles just after fertilization using TriReagent in accordance with the manufacturer's instructions (Sigma). The small RNA cDNA libraries were generated from 500 to 800 μg of total RNA as described previously [45]. Briefly, small RNA from 18 to 28 nt were isolated by acrylamide gel size fractionation, purified and ligated sequentially to 5' and 3' RNA/DNA adapters (5'adapter: 5'TGGGAATTCCTCACTrArArA3', 3'adapter: 5'P-rUrUrUCTATCCATGGACTGTidT3' from Dharmacon). Reverse transcription and PCR amplification were performed using adapter oligonucleotides. PCR products were cloned into pGEM-T Easy vector (Promega) and transformed into competent cells. Colony PCR reactions were performed using pGEM-T Easy vector primers T7 and M13R and PCR products were sequenced.

### Sequence analysis and selection of candidate miRNAs

Automated base calling of raw sequence traces was performed with the PHRED program [46]. An in-house program using the vecscreen option of BLAST was used to eliminate poor quality sequences and remove the vector sequence. Sequences with a length between 18 and 25 bp were retained for the next steps. First, using BLASTN [47], sequences identical to rRNA, tRNA, chloroplastic or mitochondrial sequences were discarded. Among the remaining sequences, we searched for those corresponding to members of families already described in the Sanger miRNA registry [3]. To do that, sequences were considered as belonging to the same family if the length and/or sequence polymorphism between all members were less than 3 nucleotides mismatches. Sequences that were not identical to a previously described miRNAs and did not belong to an already known miRNA family were mapped *in silico *by BLASTN on the the Rice Annotation Project DataBase assemblies [4]. Only perfect matches were considered. To identify sequences matching on transposable elements, each locus was mapped on the Rice Annotation Project DataBase assemblies [3] and RetrOryza database [16]. Sequences co-localising with transposable elements were discarded. We then searched for a potential miRNA* sequence using WU-BLAST [45] with a maximum distance of 800 bp and a minimum of 75% of sequence identity between the miRNA and the miRNA*. The predicted fold back structures were confirmed using RNA fold program from Vienna using default parameters [48].

Full length cDNAs corresponding to predicted miRNA precursors were searched using BLASTN against full length cDNAs deposited into GenBank. Finally the genomic location of all predicted miRNA candidates were subsequently revised by matching to the IGSRP 4 from Rice Annotation Project Database RAP-DB, which is the most comprehensive database on rice genome annotation [4].

### Selection of miRNA target candidates

Putative miRNA target sequences were predicted by two approaches. We first used a BLASTN analysis on RAP-DB [4] selecting for miRNA/mRNA interactions allowing for 4 G.U wobble and 3 mismatches, excluding those in critical positions 10 and 11, no more than 2 mismatches between nucleotides 2 and 12, and no clusters of 2 mismatches on the 3' end of the miRNAs [24]. In the second approach we used miRU program [25] against the TIGR rice genome mRNA dataset (OSA1 release 3).

### RNA gel blot analysis

Total RNA was extracted using TriReagent (Sigma) according to the manufacturer's instructions from several organs of wild type Nipponbare (leaves of young and adult plants, immature panicles, panicles just after fertilization and roots of adult plants) and from adult leaves of *osdcl1IR *lines [21]. Northern blot hybridisations were performed with 50 μg of total RNA separated in a 15% polyacrylamide gel and transferred to a Zeta-Probe GT nylon membrane [45]. Membranes were cross-linked under UV-trans-illumination and hybridised overnight at 40°C with ^32^P oligonucleotide probes complementary to the miRNA sequences. As a loading control, each blot was hybridised with a 31 nucleotides oligonucleotide labelled probe complementary to U6 snRNA. Membranes were exposed to phosphor screen (Amersham Bioscience).

### Transient expression assay in *Nicotiana benthamiana *leaves

Transient expression of rice stem-loop predicted sequences in *Nicotiana benthamiana *leaves [21] was made using the pC2300 overexpression vector, a derivative of pCambia2300 carrying Gateway BP cassettes between the maize Ubiquitine promoter (Ubi) and the Nopaline synthase 3' polyadenylation region [22]. Rice genomic fragments of ~500 bp encompassing the stem-loop predicted structure carrying the miRNA/miRNA* plus 150 nucleotides of genomic sequences flanking the 5' and 3' region were amplified by PCR and cloned in pC2300. A fragment carrying a Miniature Inverted Repeat Transposable Element (MITE) (ORSgTEMT01701966) was used as a negative control. The resulting plasmids were transferred to the *Agrobacterium *strain EHA105 by electroporation and prepared for infiltration. Two to four-week-old *Nicotiana benthamiana Rdr6IR *leaves were infiltrated [21]. Four days post-infiltration, total RNA was extracted and subjected to Northern blot as described previously.

### 5'RACE analysis

Poly (A) RNAs from total RNA rice leaves were isolated using mRNA isolation kit (Promega). An RNA adapter was directly ligated to 100 ng of mRNA using a T4 RNA ligase (Promega). Ligated mRNA were reverse transcribed using a Stratascript reverse transcription kit and with random hexamers according to the manufacturer's instructions (Stratagene). 5' Rapid Amplification of cDNA Ends (5'RACE) were performed on Os02g36924 and Os04g48390 transcripts corresponding to candidate miRNA putative targets. Initial PCR reactions were done with an adaptor primer and complementary gene specific primers. The nested PCR were performed on 1 μl of initial reactions with the same adaptor primer and complementary gene specific internal primers. The resulting PCR products were gel purified, cloned in pGEMt-easy vector (Promega) and sequenced.

### Circular RT-PCR

This was done as described [20]. Essentially total RNA from rice panicle were self ligated with T4 RNA ligase 1 (New England Biolabs). Briefly, 10 μg of RNA was incubated overnight at 14°C with 80 u T4 RNA ligase 1 in a 100 μl final volume. Following ligation, RNAs were phenol/chloroform extracted and precipitated with ethanol. Then miRNA precursors were amplified by RT-PCR with the gene specific primers:

preosa-miR1874: Reverse: AGGCACATCGAAGATTCGAACC,

Forward:TAGGGTACAGTTGGATCCCATAT

preosa-miR827a: Reverse TTCTCCAATCTCCAGGCATGCAC,

Forward CAGTCGGCTTATTGGCTCTTGG.

Only reverse primers were used in the reverse transcription assay. PCR products were resolved by gel electrophoresis and cloned into pGEM-T-easy (Promega) for sequencing.

### Cloning of osa-miR159a.2 precursor from distinct rice species and maize

Genomic fragments containing the pre-osa-miR159a.2 sequences from *Oryza coartacta *and *Oryza ridleyi *were directly identified by BLASTN using osa-MIR159 stem-loop sequence from Nipponbare cultivar against the BAC ends sequences from 12 Oryza available from OMAP project species . The *O. officinalis*, *O. alta *and *O. grandilumis *sequences were amplified by PCR from the corresponding genomic DNAs, using primers Forward (5'GGTGGTCATGGACTCATTGG'3) and Reverse (5'GCAAAGAAGAGCCGAAAGAG '3) Primers. These were designed based on conserved regions at the 5' and 3' extremities of hairpin structures identified by alignment of the six osamiR159 precursor from *Oryza sativa *ssp *japonica *cv Nipponbare. This led us to amplify genomic sequences from the indicated rice species. All sequences were confirmed by sequencing.

The maize orthologue sequence to miR159-osa-miR159a.2 from rice was identified by BLAST search of the NCBI maize EST collections (dbEST database) with a fragment of the osa-miR159a. The sequences showing highest similarity (E-value ~0) were selected and assembled with cap3 software [49]. Dotter [50] and Muscle [51] programs were used for sequence comparative analysis of cDNA sequences from rice and maize.

The structure of the putative maize pre-miR159-osa-miR159a.2 was calculated with RNA fold program from Vienna using default parameters [48].

### Locus position and nomenclature

For all our genes we used the RAP DB annotation on IRGSP4 built 4. The different osa-miR1428 locus, shown in Figure 3 correspond to:

chr3a: chromosome 3, position 23836656, 23837655

chr3b: ........................................ 33776556, 33780919

chr3c: .................. ............ 21873421, 21874420

chr1a: chromosome1, ............. 13303032, 13304031

chr1b:..................,............. 27046775, 27047774

chr7a: chromosome 7, .............18821020,18822019

chr7b: ............ .................... 5483028, 5484027

chr10: chromosome 10,............. 3939954,3940953

chr5: chromosome 5, ............18953403,18954402

chr2: chromosome 2, ...............2534585,2535584

chr9: chromosome 9, ..............18309075,18310074

## Authors' contributions

YS and ME designed the research. SL and HN constructed the small RNA libraries and performed some of miRNA expression analysis and transient expression of miRNA precursors *Nicotiana benthamiana*. CS mapped the genomic loci, predicted the targets, made RACE analysis for target cleavages and tested expression of several miRNA locus. BP contributed to bioinformatic analysis of small RNA libraries. JH, EG, JCB, CBR and MB contributed to cloning and expression analysis of miRNAs. DD cloned the pre-miRNA by cRT-PCR. CBR, OP and WK contributed to bioinformatics and experimental analysis to identify miRNA homologs different in rice species and maize. XFC produced and provided the *osdcl1 *IR lines seeds.

## Supplementary Material

Additional file 1 **Genomic locations and organizations of osa-miR827a and osa-miR1874 genes.** The genomic locations and organizations of osa-miR827a and osa-miR1874 are shown schematically in panels A and B, respectively. Click here for file

Additional file 2 **Predicted targets for osa-miR2055, osa-miR1428e, and osa-miR1874.** Predicted targets for osa-miR2055, osa-miR1428e, and osa-miR1874 are shown in panels A, B and C, respectively. Red and blue characters represent the sequences of putative targets and miRNAs, respectively. Click here for file
