# Supplementary figures and images for: Identification of precursor transcripts for 6 novel miRNAs expands the diversity on the genomic organisation and expression of miRNA genes in rice
Source: BMC Plant Biol. 2008 Dec 2;8:123. doi: 10.1186/1471-2229-8-123 (PMC2607281; doi:10.1186/1471-2229-8-123)

A

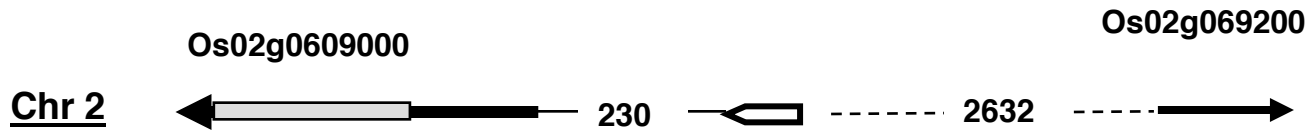

B

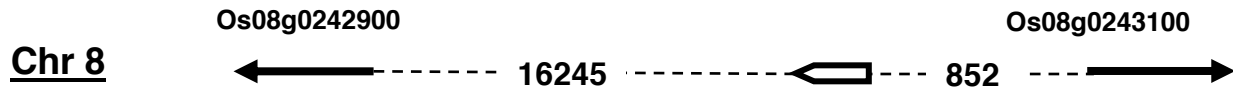

Supplement: Additional file 1 — Genomic locations and organizations of osa-miR827a and osa-miR1874 genes. The genomic locations and organizations of osa-miR827a and osa-miR1874 are shown schematically in panels A and B, respectively. [file 1471-2229-8-123-S1.pdf]
