# Supplementary material for: Identification of precursor transcripts for 6 novel miRNAs expands the diversity on the genomic organisation and expression of miRNA genes in rice
Source: BMC Plant Biol. 2008 Dec 2;8:123. doi: 10.1186/1471-2229-8-123 (PMC2607281; doi:10.1186/1471-2229-8-123)
Supplement: Additional file 2 — Predicted targets for osa-miR2055, osa-miR1428e, and osa-miR1874. Predicted targets for osa-miR2055, osa-miR1428e, and osa-miR1874 are shown in panels A, B and C, respectively. Red and blue characters represent the sequences of putative targets and miRNAs, respectively. [file 1471-2229-8-123-S2.pdf]

A

Os01g0871200

Zn Finger

```
5'-GGAACUACCUUCUCAAGAAAA-3'
    *:**********:**** ***
    CUUUGGUGGAAGGGUUCCUUU
```

Os06g0484800

RdDNA pol

```
GAAAGAACCUUCCCAGGGAAA
 ***   *********:*****
CUUUGGUGGAAGGGUUCCUUU
```

Os04g0629300

DEAD/DEAH Helicase

```
CAAACAAACUUCCUAAGGAAA
 **** * *************
CUUUGGUGGAAGGGUUCCUUU
```

Os05g0560400

F-box

```
5'-GGAACCACUGUCUUAAGGAAC-3'
   ********: **::******
   CUUUGGUGGAAGGGUUCCUUU
```

Os01g0323600

AdoMet Synthetase

```
5'-GAAGGCAUCUUCCUGAGGAAG-3'
   ***: **:*****::*****:
   CUUUGGUGGAAGGGUUCCUUU
```

B

Os03g0289100

Protein kinase

```
5'-CAAAUACAAGGCAUUAUCUUC-3'
   ***** ** ***********
3'  GUUUAAGUACCGUAAUAGAAU
```

C

Os04g0311100

Hyp protein

```
5'-UGGCAACACCUCCAUCCAAU-3'
    **   *************
   CCCAAUGUGGAGGUAGGUAU
```

Os10g0337400

Protein kinase

```
5'-GUGUGAUCCCUCCAUCCAUU-3'
   * ** ** ***********
   CCCAAUGUGGAGGUAGGUAU
```
